# Photosynthetic Electron Flows and Networks of Metabolite Trafficking to Sustain Metabolism in Photosynthetic Systems

**DOI:** 10.3390/plants13213015

**Published:** 2024-10-28

**Authors:** Neda Fakhimi, Arthur R. Grossman

**Affiliations:** 1 Department of Biosphere Sciences and Engineering, The Carnegie Institution for Science, 260 Panama Street, Stanford, CA 94305, USA; agrossman@carnegiescience.edu; 2 Courtesy Appointment, Department of Biology, Stanford University, Stanford, CA 94305, USA

**Keywords:** photosynthetic/respiratory electron flow, chloroplast, mitochondria, transporters, sugar phosphates, dicarboxylic acids, carbon concentrating mechanism, photorespiration, fermentation

## Abstract

Photosynthetic eukaryotes have metabolic pathways that occur in distinct subcellular compartments. However, because metabolites synthesized in one compartment, including fixed carbon compounds and reductant generated by photosynthetic electron flows, may be integral to processes in other compartments, the cells must efficiently move metabolites among the different compartments. This review examines the various photosynthetic electron flows used to generate ATP and fixed carbon and the trafficking of metabolites in the green alga *Chlamydomomas reinhardtii*; information on other algae and plants is provided to add depth and nuance to the discussion. We emphasized the trafficking of metabolites across the envelope membranes of the two energy powerhouse organelles of the cell, the chloroplast and mitochondrion, the nature and roles of the major mobile metabolites that move among these compartments, and the specific or presumed transporters involved in that trafficking. These transporters include sugar-phosphate (sugar-P)/inorganic phosphate (Pi) transporters and dicarboxylate transporters, although, in many cases, we know little about the substrate specificities of these transporters, how their activities are regulated/coordinated, compensatory responses among transporters when specific transporters are compromised, associations between transporters and other cellular proteins, and the possibilities for forming specific ‘megacomplexes’ involving interactions between enzymes of central metabolism with specific transport proteins. Finally, we discuss metabolite trafficking associated with specific biological processes that occur under various environmental conditions to help to maintain the cell’s fitness. These processes include C4 metabolism in plants and the carbon concentrating mechanism, photorespiration, and fermentation metabolism in algae.

## 1. Introduction

Photosynthetic eukaryotes have evolved a network of complex, integrated metabolisms that occur in distinct subcellular compartments [1,2]. These compartments are usually bounded by lipid membranes, house-specific pathways, and manage intracellular trafficking of metabolites, reducing equivalents, and proteins. Subcellular compartmentation may confine specific metabolites or processes that can have toxic or detrimental effects if more widely distributed throughout the cells. *Chlamydomonas reinhardtii* (designated *Chlamydomonas* throughout) is a well-studied, unicellular photosynthetic green alga that is a flagship organism of the Department of Energy and serves as a model for studies of photosynthesis, cilium/flagella biogenesis and function, the dissection of various metabolic pathways, and their integration and control. Two distinct interacting organelles that house a variety of metabolic processes are chloroplasts and mitochondria [3,4]; these compartments take up and synthesize metabolites that can be distributed within cells. They have their own DNA (and DNA replication machinery), synthesize transcripts and polypeptides, and assemble proteins and their cofactors into complexes that function in photosynthetic and respiratory metabolism, while also impacting intracellular redox and energetic conditions that can tune and integrate specific physiological/metabolic processes [5,6]. These organelles also perform biosynthetic functions that generate retrograde signals that reflect their energetic and metabolic status; such signals can impact nuclear gene expression, protein synthesis and modification, and the assembly and activity of protein complexes [7,8,9]. Other cellular compartments, including the glyoxysomes/peroxisomes, Golgi bodies, and endoplasmic reticulum (ER), are also critical for various physiological processes, including lipid metabolism, detoxification of reactive oxygen species (ROS), protein maturation and trafficking, post-translational modifications, delivery of material to the extracellular space, and intracellular signaling [10,11,12,13,14,15]. In this review, we discuss the various electron flows and the transport of carbon and redox equivalents across the chloroplast envelope, interactions between chloroplasts and mitochondria, and how such interactions impact the physiology of the cells. The work presented will focus on *Chlamydomonas*, although we also discuss vascular plants and other systems to highlight specific points.

## 2. Subcellular Compartments

Metabolic pathways are often interconnected and reliant on the exchange of precursor metabolites among different subcellular compartments. To enable and regulate metabolite movement across boundaries that delineate cellular compartments, specific translocators/transporters (we use the term transporter throughout) are essential to manage the flow of small, often energetic molecules among the compartments, which is integral to connectivity among the cell’s metabolic networks [16,17]. As a wide variety of molecules synthesized in one cellular compartment are required in other locations in the cell, photosynthetic eukaryotes have evolved a diverse suite of metabolite transporters that participate in distributing metabolites among the cell’s compartments, although our understanding of such trafficking remains limited.

**Figure 1:**
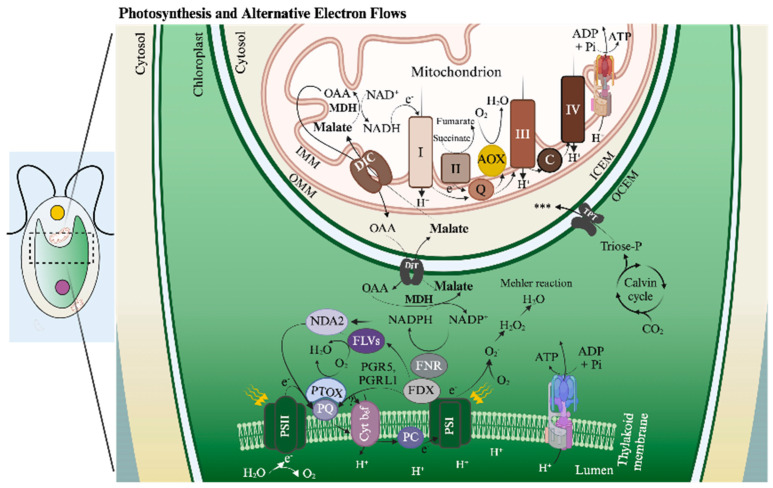
Photosynthetic and respiratory electron transport and their interactions. Linear photosynthetic electron transport (bottom) involves excitation of reaction centers, photosystem I and II, (PSI, PSII) and extraction of electrons from H_2_O by the PSII O_2_ evolving complex. Extracted electrons pass through PSII reaction centers, the plastoquinone (PQ) pool, Cytochrome b_6_f (Cytb_6_f), plastocyanin (PC) and to PSI where they are used to generate reduced ferredoxin (FDX) and NADPH through the activity of Ferredoxin NADP^+^ reductase (FNR); the NADPH and the ATP synthesized by the ATP synthase (fueled by the proton gradient across thylakoid membranes) and the NADPH are used to reduce CO_2_ and drive metabolic processes in the cells. Reducing electrons generated on the acceptor side of PSI or PSII can be routed through AEF pathways: Cyclic electron flow occurs through both PGR5/PGRL1, and the NDA2 pathways. In the Mehler reaction, PSI-derived electrons are used to reduce O_2_, and the ROS generated (O_2_^−^ and H_2_O_2_) can be converted to H_2_O through superoxide dismutase and catalase/ascorbate peroxidase. In pseudocyclic electron flow (PCEF), electrons from PSI/FDX are transferred to the diiron proteins (FLV) to reduce O_2_ to H_2_O. The plastoquinol terminal oxidase (PTOX) catalyzes the reduction of O_2_ on the acceptor side of PSII. In chloroplast-to-mitochondria electron flow (CMEF), electrons are exported from the chloroplast to the mitochondrion through the function of OAA (or 2-oxoglutarate)/malate redox shuttles on both the chloroplast and mitochondria envelopes; reductant is shuttled between the compartments through the interconversion of malate/NAD(P)^+^ to OAA/NAD(P)H. ***, Transporting triose-P out of chloroplasts is another potential avenue for delivering reductant to mitochondria. However, since there is still little reported evidence for that route of delivery, the extent to which it provides reductant to power mitochondrial respiration is uncertain. The electrons released from these reductants are used to drive oxidative phosphorylation in mitochondria (top) through either cytochrome oxidase (complex IV) or alternative oxidases (AOXs), generating additional ATP. ICEM, inner chloroplast envelope membrane; OCEM, outer chloroplast envelope membrane; IMM, inner mitochondrion membrane; OMM, outer mitochondrion membrane. Representation of the photosynthetic electron transport chain was modified from Grossman in [18]. Created with BioRender.com.

**Figure 2:**
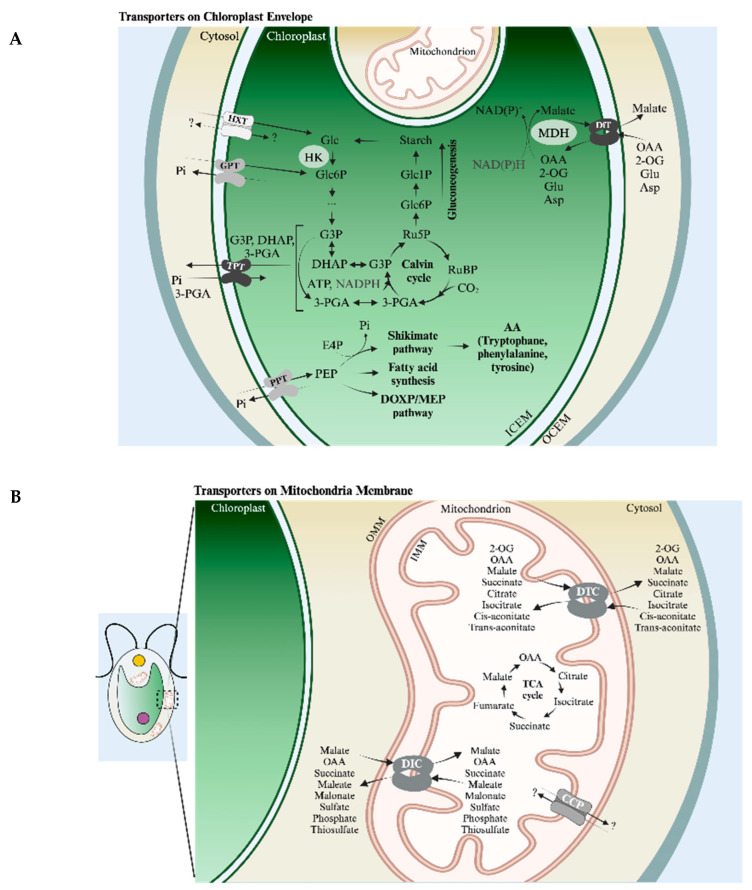
Overview of characterized and uncharacterized major fixed carbon and reductant transporters on inner chloroplast and mitochondrial envelope membranes. (**A**) Transporters on the chloroplast envelope in *Chlamydomonas* are divided into two major groups: sugar-P/phosphate transporters, including GPTs, TPTs, and PPTs, which transport both fixed carbon and reductants, and dicarboxylate transporters, which mainly shuttle reductants. Based on Doebbe et al. [19], there might also be a hexose transporter on the chloroplast envelope, although the specificity and the mechanism of transport have not been elucidated. While the substrates transported are indicated, many of these bidirectional sugar-P transporters can use multiple substrates (and counter-substrate); Pi is considered a preferred counter-substrate. (**B**) Transporters on the mitochondrial inner membrane of plants, with analogous proteins in *Chlamydomonas*), include di- and tricarboxylate transporters. Most of the metabolites translocated across the mitochondria membrane are associated with the TCA cycle. Reductant imported into the mitochondria can drive oxidative phosphorylation. CCPs (two in *Chlamydomonas*) are mitochondrial transporters that increase in abundance (transcript level) under low CO_2_ conditions, but their functions are yet to be determined. Dark gray indicates that some transporters from this category have been characterized in *Chlamydomonas*, while light gray indicates that the transporter has not yet been characterized; the presence of these transporters on the mitochondrial envelope of *Chlamydomonas* and their contribution to its metabolism are predicted from information derived from plant system. DIC, dicarboxylate translocator on the mitochondrial envelope; DiT, dicarboxylate translocator on the chloroplast envelope; DTC, tricarboxylate translocator; GPT, glucose 6-phosphate/phosphate transporter; HK, hexokinase; HXT, hexose transporter; MDH, malate dehydrogenase; PPT, phosphoenol pyruvate/phosphate transporter; TPT, triose-phosphate/phosphate transporter; DOXP/MEP, 1-deoxyxylulose 5-phosphate/2-C-methylerythritol 4-phosphate pathway; TCA, tricarboxylic acid cycle; AA, amino acid; ATP, adenosine triphosphate; Asp, aspartate; CO_2_, carbon dioxide; DHAP, dihydroxyacetone phosphate; E4P, D-erythrose 4-phosphate; Glc, glucose; Glc1P, glucose 1-phosphate; Glc6P, glucose 6-phosphate; G3P, glyceraldehyde-3-phosphate; Glu, glutamate; 3-PGA, 3-phosphoglyceric acid; NAD(P)(H), nicotinamide adenine dinucleotide (phosphate); OAA, oxaloacetate; 2-OG, 2-oxoglutarate; PEP, phosphoenol pyruvate; Pi, inorganic phosphate; RuBP, ribulose 1,5-bisphosphate; Ru5P, ribose-5-phosphate; ICEM, inner chloroplast envelope membrane; OCEM, outer chloroplast envelope membrane; IMM, inner mitochondrion membrane; OMM, outer mitochondrion membrane. Created with BioRender.com.

Much of the work discussed in this review focuses on chloroplasts and mitochondria. A major chloroplast function is the photosynthetic production of fixed carbon, which involves generating ATP (chemical bond energy) and reductant [NADPH and reduced ferredoxin (FDX)] by light-driven, photosynthetic electron transport (PET), as depicted in Figure 1 (bottom). The Calvin–Benson cycle (CBC) uses these products to fix/reduce inorganic carbon (Ci, which includes CO_2_, HCO_3_^−^, CO_3_^−2^) in the form of CO_2_, which is initially bonded to ribulose 1,5-bisphosphate (RuBP) by the enzyme ribulose 1,5-bisphosphate carboxylase (Rubisco); the Rubisco reaction generates two molecules of 3-phosphoglycerate (3-PGA) which are reduced to triose phosphate [triose-P includes glyceraldehyde 3-phosphate (G3P) and dihydroxyacetone phosphate (DHAP)] with the fixation of a single CO_2_ molecule. The fixed carbon can be used for growth and development, which is dependent upon biosynthetic processes occurring throughout the cell. Additionally, mitochondria can use photosynthetically generated fixed carbon and reductant to drive oxidative phosphorylation and ATP synthesis. While mitochondria mostly use O_2_ as a terminal electron acceptor, the proteins that facilitate the transfer of electrons to O_2_ can be either cytochrome oxidase (CytOx) or alternative oxidases (AOXs). Figure 1 (top) shows the passage of electrons through the three mitochondrial electron transport complexes and cytochrome c before reaching CytOx. In contrast, AOX accepts electrons from the respiratory quinone pool, which is reduced by respiratory complex I and II. This difference in mitochondrial electron flow results in a difference in the energy generated by the passage of an electron through the system. This figure also shows the potential trafficking of electrons generated by PET and respiratory metabolism, primarily through the activity of the malate shunt, which is discussed in Section 4.3.2 and Section 4.3.3. A more comprehensive view of transporters associated with the chloroplast and mitochondrial membranes (many of which have not been identified) is shown in Figure 2A,B, respectively; their contributions to metabolite exchange are discussed in several sections of this review.

## 3. Photosynthetic Electron Flows, ATP Production, and Quenching Excess Absorbed Light Energy

Linear and alternative photosynthetic electron flows (LEF and AEF, respectively) and the quenching of excess absorbed light energy all impact the fixation of CO_2_, the generation of energy/ATP in chloroplasts, and the movement of redox equivalents and fixed carbon to other cellular compartments [18]. These electron flows can also lessen damage caused by ROS formation when photosynthetic organisms absorb more light energy than they can readily use to fuel photosynthetic CO_2_ fixation. The absorption of excess light can occur during midday as light intensities become high, and at lower light intensities if growth becomes limited because of suboptimal abiotic conditions, including limiting access to nutrients and fluctuations in ambient temperatures; as abiotic conditions become suboptimal, lower-intensity light saturates photosynthesis, and more of the absorbed light energy cannot be productively used for growth and anabolic processes [20,21,22].

Excess absorbed excitation can be eliminated by both nonphotochemical quenching (NPQ) and photochemical quenching. NPQ and, in particular, qE (energy dependent quenching), prominent in many photosynthetic organisms, require a proton gradient across the thylakoid membranes and the accumulation of zeaxanthin [23,24]. It promotes the dissipation of excess absorbed light energy as heat through pigment–protein complexes that harbor specific subunits with homology to light-harvesting antennae proteins. These proteins include PSBS in plants [25,26] and LHCSR1 and LHCSR3 [27,28,29] in certain algae (e.g., *Chlamydomonas*). Other modes of NPQ exist, including state transitions, which involve the movement of antenna between the two photosystems (qT) [30,31], photoinhibition (qI) [32], and zeaxanthin-independent quenching [33].

Absorbed light energy can also be productively used and safely eliminated (when in excess) by various forms of PET and photochemical quenching through AEF. LEF generates chemical bond energy in the form of ATP (as well as reductant) but does not provide enough ATP to sustain optimal levels of photosynthesis/CO_2_ fixation. Various AEFs can produce the additional energy needed for increasing the efficiency of CO_2_ fixation. Among these AEF pathways is cyclic electron flow (CEF) around photosystem I (PSI) [34,35], which generates a proton gradient across the thylakoid membranes that fuels ATP production, and various routes for the passage of electrons to O_2_, resulting in the generation of H_2_O in what is commonly referred to as H_2_O-to-H_2_O cycles [36,37]. These pathways include (*i*) Mehler-type reactions (noncatalyzed reduction of O_2_ by PSI) [38,39,40], (*ii*) flavodiiron oxidoreductase (FLV) reduction of O_2_ in a process designated pseudocyclic electron flow (PCEF) [41,42,43,44,45], (*iii*) plastoquinol terminal oxidase (PTOX) activity [46,47,48], commonly referred to as chlororespiration [47,49,50,51], and (*iv*) chloroplast to mitochondria electron flow (CMEF) [42]. CMEF involves the movement of a photosynthetically generated reductant to the mitochondrial electron transport chain, where it can be used to reduce O_2_ via either CytOx [52,53] or AOXs [54,55]. CMEF is further discussed in Section 4, which describes the triose-P/phosphate and the oxaloacetate/malate shuttles. ATP generated by AEF pathways supplies the energy in excess of what is produced by LEF to satisfy the ATP requirement for efficiently fixing CO_2_ and also can ameliorate the damaging effects of excess absorbed excitation. These electron flow reactions are depicted in Figure 1 (bottom) and discussed below.

Several studies have examined the mechanisms associated with CEF, for which there are two major pathways; one involves PGR5/PGRL1 [56,57,58] and the other involves NAD(P)H-plastoquinone (PQ) reductase, NDH1 in plants and NDH2 (NDA2) in algae like *Chlamydomonas* [59,60,61,62]. Various studies have also characterized the Mehler reaction [38], which can generate a ΔpH across thylakoid membranes, enabling ATP synthesis and the establishment of NPQ [40], although Mehler activity can also generate damaging ROS, including superoxides (O_2_^−^) and hydrogen peroxide (H_2_O_2_). PCEF, like the Mehler reaction, involves PSI activity, but the electrons are transferred from PSI to FLVs, likely through FDX [63], which may then reduce O_2_ directly to H_2_O [45]. This reaction occurs in cyanobacteria, green algae, mosses, liverworts, and gymnosperms but has not been observed in angiosperms; it appears especially relevant to the maintenance of photosynthetic activity, both immediately after the cells experience a dark period, when the CBC is not fully activated, and under fluctuating light conditions [64]. In contrast to the Mehler-type reactions and PCEF, PTOX/chlororespiration uses electrons that accumulate in the photosynthetic PQ pool to reduce O_2_ to H_2_O [65]. The hyper-reduction of the PQ pool can be a consequence of photosystem II (PSII) activity, the limited availability of electron acceptors downstream of the PQ pool in PET, or in the Calvin Benson cycle, and the transfer of electrons to the PQ pool from NAD(P)H via NDH1 or NDH2 (NDA2). PTOX appears to manage the redox state of the PQ pool, protecting cells from PQ over-reduction during stress, which can elicit PSII charge recombination and the generation of reactive singlet O_2_ (^1^O_2_). *Chlamydomonas* has two PTOX isoforms (PTOX1, PTOX2) [47] with distinct functions. PTOX2 may be important for controlling the redox state of the PQ pool, whereas PTOX1 appears to function in PQH reoxidation for supporting phytoene desaturase activity [66].

AEF can also involve the movement of a reductant among cellular compartments. Metabolic connectivity between chloroplasts and mitochondria (e.g., CMEF) was suggested decades ago based on studies using inhibitors of mitochondrial electron transport and mutants in the chloroplast ATP synthase [67] and respiration [68]. Prominent reductant/fixed carbon shuttles are associated with CMEF and include dicarboxylate transporters, such as the oxaloacetate (OAA)/malate transporters (OMT), the sugar-P and, in particular, the triose-P/phosphate transporters (TPT) [69,70,71] (Figure 1 and Figure 2A), and the malate-aspartate shuttle [72,73,74]; the last of these in *Chlamydomonas* is associated with the movement of the reductant to the glyoxysome.

The relative importance of the different AEF pathways and shuttles for synthesizing supplemental ATP to support CO_2_ fixation and managing excess excitation are still uncertain, although they are likely to be impacted by nutrient conditions, light levels, and the cellular redox/energetic state. In a recent study, the potential for energy production by these pathways was evaluated based on analyses of mutants and the administration of inhibitors of electron transport. The results suggest that CEF, PCEF, and CMEF can each provide a large fraction of the energy needed to sustain photosynthesis, with the most energetically efficient pathway being CMEF [42]. These pathways exhibit compensatory activities; when the function of one is compromised, the others can take over and provide enough energy to drive maximal or near-maximal photosynthesis. Additionally, while it has been reported that combinations of AEF pathways can produce enough ATP to make up the LEF energetic deficit, the activities measured in cells lacking one or more of these pathways may not accurately reflect the in vivo proportions of these activities, since quantifications using either mutants and/or pathway inhibitors would likely alter the contributions of the remaining pathway(s). We are still in the early stages of elucidating energetic contributions of AEF pathways in vivo, the polypeptide subunits integral to pathway functions, the assembly of these subunits into megacomplexes, the ways in which the different AEF activities are regulated and ‘communicate’ with each other, and the paths along which they evolved to become integral for certain organisms but not for others.

## 4. Chloroplast and Mitochondrial Transporters of Fixed Carbon and Reductant

### 4.1. Overview

Efficient, controlled metabolite transport (e.g., products of photosynthesis) and exchange between subcellular compartments and among cells are critical for the fitness of organisms as they experience a varied and dynamic spectrum of environmental conditions. Chloroplasts and mitochondria, the energy ‘giants’ of photosynthetic eukaryotes, perform transcription, have their own ribosomes that actively translate proteins, and play crucial roles in several metabolic pathways, including respiratory and photosynthetic metabolism, nitrogen, phosphorus and sulfur assimilation, glycolysis, the tricarboxylic acid (TCA) cycle, and the biosynthesis of fatty acids, amino acids, heme/chlorophyll and terpenoids/carotenoids. While a range of analyses have explored the functions of specific metabolite transporters in photosynthetic eukaryotes [reviewed in [1,72,75]], most of this work has been performed with vascular plants, with fewer studies on algae.

As mentioned above, the prominent shuttles for trafficking fixed carbon and reductant from/into chloroplasts and mitochondria include sugar-P transporters and di- and tricarboxylate transporters (antiporters) (DiT, DIC, DTC; most commonly OMT) [1,44,69,70,71,72] (Figure 2A,B). Also important for partitioning the reductant are the malate dehydrogenase enzymes (MDH), which catalyze the reductive interconversion of OAA and malate, and glyceraldehyde 3-P dehydrogenase (GAPDH), which can reduce 3-PGA to G3P that can then be isomerized to DHAP. These reductant trafficking mechanisms have a dominant role in CMEF. Photosynthetic organisms have multiple OMTs and sugar-P transporters that are integral to various cell membranes (especially chloroplast, mitochondria, peroxisomes). As discussed, the reductant generated in chloroplasts can move to mitochondria, where it can serve as a substrate for respiratory electron transport through CytOx and/or AOX. Electrons directed toward AOX [42,54,55] are able to generate some ATP through the formation of an electrochemical potential (AOX itself does not pump protons) across the mitochondrial membranes but not as much as is generated by the CytOx pathway. AOX can also be thermogenic, is cyanide insensitive, enables continued operation of glycolysis and the TCA cycle under stress conditions when there may be restricted electron flow through CytOx, helps balance C:N, ATP:ADP, NAD(P)H:ATP ratios, and limits hyper-reduction of the respiratory chain and the accumulation of ROS [54,76,77,78].

Major metabolite-carbon transporters associated with chloroplasts and mitochondria are the sugar-P/Pi transporters (antiporters), generally referred to as phosphate transporters (PTs), and non-phosphorylated metabolite transporters that include the di- and tricarboxylate transporters. The direction of transport is driven by substrate concentrations. The sugar-P transporter family represents a suite of prominent transporters that belong to the major facilitator superfamily (MFS), enabling translocation of small molecules in response to ion gradients across cellular membranes [79]. In *Chlamydomonas*, most of the sugar-P transporters have been designated TPT and are part of the TPT family of proteins. TPTs (in this general sense) of plant and algal chloroplasts, and potentially mitochondria envelope membranes, assume a critical role in regulating phosphate and fixed carbon metabolism, with the plastid translocators mostly exporting photosynthetically fixed carbon [71,80], at least during the day. The TPTs are also referred to as organo-P/phosphate antiporters [70] and, when associated with the plastid envelope, can be designated plastid PTs. Some transporters in the TPT family may be most specific for triose-P (G3P, DHAP)/phosphate exchange, although various members of this family also facilitate the exchange of other phosphorylated sugars, including glucose 6-P (GPTs) [81], pentose-P or Xul5P (XPT) [82], and phosphoenolpyruvate (PEP) (PPT) [83].

Overall, these shuttles assume a central role in integrating/regulating carbon metabolism with the availability/metabolism of other nutrients. More complex organic products of photosynthesis such as fatty acids and isoprenoids are also synthesized in, and can be transported out of, chloroplasts [84,85,86,87].

### 4.2. Chloroplast Sugar-P Transporters

#### 4.2.1. Introduction

In plants, the photosynthetic fixation of CO_2_ leads to the synthesis of sugar-Ps, which can be exported to the cytoplasm and converted to sucrose [88]. The sugar-Ps and sucrose can fuel metabolic processes in other tissues/organs [89,90] and compartments in plants, including the mitochondria, ER, and peroxisomes/glyoxysomes [71,91]. The fixed carbon can also drive the synthesis of starch and triacylglycerol, major plant and algal storage carbon compounds [92]. The distribution of fixed carbon among the different plant tissue types involves the transport of sugars (sucrose) through the phloem.

Phylogenetic examination of 652 plastid PT genes across 101 diverse algal and land plant genomes identified several transporters in the TPT family, including GPT, TPT, XPT, and PPT; they are present in all plant and algal species examined. This family of transporters evolved prior to the divergence of red and green algae. Additionally, XPTs originated from duplications of GPTs that arose during the foundational stages of the streptophyte lineage [80]. In *Arabidopsis*, there are 16 plastid PT genes, including a single copy of a *TPT* (At*TPT1*), specifically involved in triose-P transport, two *PPTs*, one *XPT*, and two *GPT*. The *Arabidopsis* TPT1 protein was shown to be chloroplast localized based on imaging of cells expressing an AtTPT1-GFP (green fluorescence) fusion protein. Additionally, *Arabidopsis* has a relatively large number of truncated *PPT* (six) and *GPT* (four) genes with nearly identical intron/exon architecture compared to their functional counterparts [93,94].

In *Arabidopsis,* a primary function of the chloroplast TPT is to export triose-P from the stroma to the cytosol, exchanging it with cytosolic phosphate. This exchange is vital for maintaining optimal rates of growth and photosynthesis but is influenced by the availability of phosphate, which, in plants, is released (hydrolyzed) from triose-P during sucrose synthesis [95]. Over the course of the day, as the requirement for and the rate of sucrose synthesis decrease, there is a corresponding reduction in intracellular phosphate levels. This decrease results in reduced TPT activity and a shift in the direction of triose-P utilization away from growth and toward starch synthesis [96]. Hence, in plants, TPT also plays a role in partitioning intracellular carbon between sucrose and starch [95,97]. Recently, a role for plastid PTs in enhancing disease resistance was shown to involve the export of G3P to the cytosol. This export can trigger defense signaling pathways, including the MAPK (mitogen-activated protein kinase) pathway. These pathways, in turn, can elicit altered expressions of nuclear genes, ultimately resulting in the mediation of broad-spectrum resistance to pathogens, including viruses [98].

#### 4.2.2. *Chlamydomonas* Plastid PTs

Plastid PTs have the distinction of being the first inner chloroplast envelope membrane transporters identified at the molecular level and have been subjected to biochemical characterizations in heterologous systems [99,100]. Based on the crystal structure of a red algal (*Galderia sulphuraria*) TPT [101] and in vitro experiments using spinach chloroplasts [102], it was suggested that certain TPTs facilitate two potential reactions in the light; triose-P/phosphate (export of fixed carbon) and triose-P/3-PGA (export of reducing equivalents) exchange across the inner chloroplast envelope membrane.

BLAST searches with the AtTPT1 protein identified 32 putative *Chlamydomonas* TPTs (encoded on v6.1 genome), with four specific members, designated CreTPT10, CreTPT2, CreTPT3, and CONSERVED GREEN LINEAGE 51 (CGL51), predicted to possess a chloroplast transit peptide [71]. These TPT-homologous transporters were included in a phylogenetic analysis of plant and algal plastid PTs that suggested that CreTPT3 is most likely to use triose-P as its substrate, whereas CreTPT2 and CreTPT10 could potentially act as PPTs (use PEP). The CGL51 amino acid sequence, on the other hand, is congruent with GPT and/or XPT being its preferred substrate, as previously suggested [80].

Among the plastidic PTs, CreTPT3 and CreTPT2 exhibited the greatest identity/similarity to AtTPT1. Experiments involving protein reconstitution in liposomes demonstrated that both CreTPT2 and CreTPT3 share nearly identical substrate preferences; they are highly selective in facilitating the exchange of triose-P (DHAP) and 3-PGA with Pi [71]; this contradicts the previous suggestion, based on informatics, that TPT2 functions as a PPT. Although CreTPT3 and CreTPT2 in *Chlamydomonas* are both on the chloroplast envelope and have been shown to have similar substrate specificities, they do display distinct expression patterns, as indicated by RNA-seq [103,104] and RT-qPCR [71]. These different expression patterns suggest that they function at different times over the diel cycle. The numerous *TPT* homologues in *Chlamydomonas* are listed in Table 1.

#### 4.2.3. Mutants Lacking Proteins in the TPT Family

Various null mutants for members of the *TPT* gene family have been isolated. Under light intensities of 250–300 µmol photons m^−2^ s^−1^, the *Chlamydomonas tpt3* null mutants ceased to grow and exhibited an elevated accumulation of stored carbon/starch, triacylglcerides, and intracellular triose-P and 3-PGA. The mutants also exhibited an increased 1-qL value (reflects redox state of the PQ pool of the PET system), a decrease in PSI activity in moderate light, damage to the photosynthetic apparatus (photoinhibition), and the accumulation of ROS, particularly in the chloroplast [71]. These changes in intracellular metabolites and redox conditions resulted in reduced CBC activity, a decline in PET, and an excess of reducing equivalents in the stroma, which likely leads to a decrease in ATP and NAD(P)H accumulation and can ultimately lead to cell death.

Dramatic metabolic alterations in the *Chlamydomomas tpt3* mutant led to major changes in the ability of the cells to cope with light and the associated redox changes that light elicits; even relatively low/moderate light conditions were stressful for this mutant. The phenotypes of Cre*tpt2* mutants were much less severe (compromised at higher light intensities) than that of Cre*tpt3*, suggesting that while the two activities might be similar, *TPT3* is more active/abundant than Cre*TPT2*.

Overall, the study of *Chlamydomonas* TPT transporters by Huang et al. (2023) [71] concluded that the TPT3 translocator plays a crucial role in channeling fixed carbon out of the chloroplast to support central carbon metabolism, but it may also act as a redox shuttle for transferring reducing equivalents to the cytoplasm of the cell.

#### 4.2.4. PPT Transport

**Figure 3:**
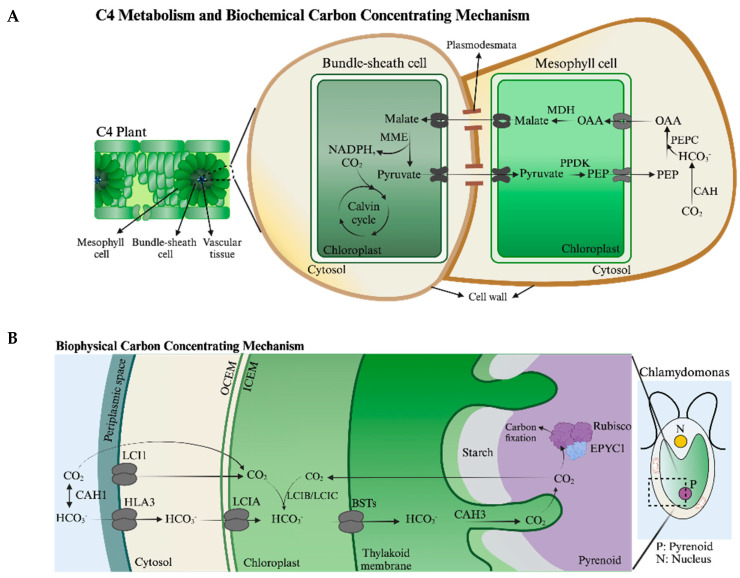

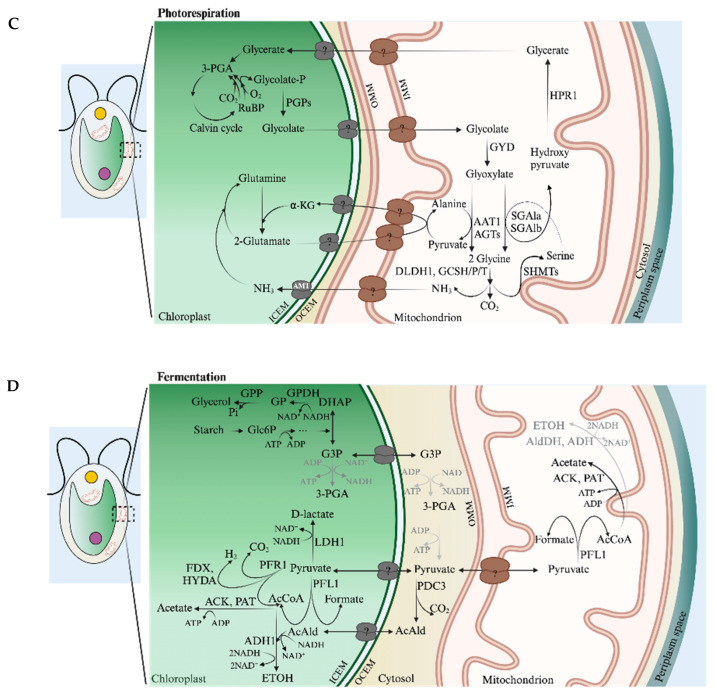
Metabolite transport required for various metabolic processes in the cell. (**A**) CO_2_-concentrating mechanism in a NADP-malic enzyme type C4 plant, such as sugarcane [adapted with some modifications from [156]]. (**B**) Trafficking of Ci across the plasma membrane and to the pyrenoid. CO_2_ either directly diffuses across the plasma membrane or is transported by LCI1. It can also be converted to bicarbonate by the activity of CAH1 and transported by HLA3, LCIA, and BSTs through the plasma membrane, chloroplast envelope, and thylakoid membranes, respectively. In the thylakoids that penetrate the pyrenoid, the bicarbonate is converted to CO_2_ through the activity of CAH3, and then fixed by Rubisco. The components and their locations in the cell are based on studies with *Chlamydomonas* [157]. (**C**) Putative metabolite transport required for photorespiration in *Chlamydomonas*. Transporters responsible for metabolite transport at the chloroplast and mitochondrial envelope membranes; most of these transporters have not yet been identified in *Chlamydomonas* (except for ammonium transporter, AMT). (**D**) Fermentation in *Chlamydomonas*. Glycerol, lactate, CO_2_, H_2_, acetate, ethanol, and formate are fermentative end-products. The succinate-producing pathway (reverse TCA cycle) is not shown. Gray arrows in the cytosol indicate omitted steps under fermentative conditions. The ethanol pathway in mitochondria has been shown only at the activity level, so it is indicated in gray. Transporters responsible for metabolite transport at the chloroplast envelope membrane and mitochondria membrane are not yet characterized [adapted from *Chlamydomonas* Source Book [158]]. AAT1, alanine aminotransferase; ACK, acetate kinase; ADH, alcohol dehydrogenase; AGT, alanine-glyoxylate transaminase; AldDH, aldehyde dehydrogenase; BST, bestrophin; CAH, carbonic anhydrase; DLDH1, dihydrolipoyl dehydrogenase; EPYC1, essential pyrenoid component 1; FDX, ferredoxin; GCSH/P/T, glycine cleavage system, H-protein/P-protein/T-protein; GPDH, glycerol-3-phosphate dehydrogenase; GPP, glycerol-3-phosphate phosphatase; GYD, glycolate dehydrogenase; HPR, hydroxypyruvate reductase; HYDA, FeFe hydrogenase; LCI, low-CO_2_ inducible protein; LDH1, lactate dehydrogenase 1; MME, malic enzyme; PDC3, pyruvate decarboxylase; PEPC, phosphoenolpyruvate carboxylase; PFL1, pyruvate formate lyase 1; PFR1, pyruvate ferredoxin oxidoreductase; PGP, phosphoglycolate phosphatase; PPDK, pyruvate phosphate dikinase; PAT, phosphate acetyltransferase; Rubisco, ribulose-1,5-bisphosphate carboxylase/oxygenase; SGA1, serine glyoxylate aminotransferase; SHMT, serine hydroxymethyltransferase; AcAld, acetaldehyde; AcCoA, acetyl coenzyme A; CO_2_, carbon dioxide; DHAP, dihydroxyacetone phosphate; ETOH, ethanol; Glc6P, glucose 6-phosphate; G3P, glyceraldehyde-3-phosphate; GP, glycerol-3-phosphate; H_2_, hydrogen; HCO_3_^−^, bicarbonate; NH_3_, ammonium; NADPH, reduced nicotinamide adenine dinucleotide phosphate; O_2_, oxygen; OAA, oxaloacetate; PEP, phosphoenolpyruvate; 3-PGA, 3-phosphoglyceric acid; Pi, inorganic phosphate; RuBP, ribulose 1,5-bisphosphate. ICEM, inner chloroplast envelope membrane; OCEM, outer chloroplast envelope membrane. Created with BioRender.com.

PEP is an intermediate in a number of cell processes, including glycolysis and C4 metabolism. It also plays a pivotal role in the shikimate pathway [105,106,107] and supports the synthesis of fatty acids [108,109,110] and isoprenoids [94]. Various biosynthetic pathways for which PEP is integral require this metabolite be transported between organelles and sometimes between cell types. For example, C4 metabolism, a mechanism for concentrating CO_2_ around Rubisco in bundle sheath cells of certain grasses such as maize and sugar cane, facilitates CO_2_ fixation and allows plants to attain higher photosynthetic efficiencies than C3 plants [e.g., ~4.3% in C4 plants relative to 3.5% in C3 plants [111]]. Additional discussion of this process is presented in Section 5.1, with the pathway for C4 metabolism depicted in Figure 3A.

The shikimate pathway is involved in the synthesis of aromatic amino acids, such as L-tryptophan, L-phenylalanine, and L-tyrosine. The initial enzymatic step of this pathway is performed using 3-deoxy-D-arabino-heptulosonate 7-phosphate synthase (DAHPS), which catalyzes an aldol condensation of PEP and D-erythrose 4-phosphate (E4P) to generate 3-deoxy-D-arabino-heptulosonate 7-phosphate (DAHP) [112]. The *Arabidopsis* genome contains three *DAHPS* genes: At*DAHPS*1 (AT4G39980), At*DAHPS*2 (At4g33510), and At*DAHPS*3 (At1g22410). Based on homology to *Arabidopsis* DAHPS, we identified a single putative *DAHPS* locus (Cre17.g726750_4532) on the *Chlamydomonas* genome. The *Chlamydomonas* protein has ~57% amino acid identity to its *Arabidopsis* homologue and is predicted to be localized to the chloroplast.

PEP supports the production of the C5 compound isopentenyl diphosphate (IPP), which is used for isoprenoid and sterol synthesis. Two distinct pathways in vascular plants can be used to synthesize these compounds. In the cytosolic compartment of the cell, sterols are produced via the mevalonate (acetate-MVA) pathway, while in plastids, isoprenoids like β-carotene, the phytyl side-chain of chlorophylls and the nonaprenyl chain of PQ are synthesized via the 1-deoxyxylulose 5-phosphate/2-C-methylerythritol 4 phosphate pathway (DOXP/MEP pathway) [113,114]. Although the acetate-MVA pathway generally contributes to prenyl sidechain biosynthesis for vascular plant mitochondrial ubiquinones [115,116], *Chlamydomonas* and other Chlorophyta [117] utilize the DOXP/MEP pathway for synthesizing plastidic isoprenes, as well as for the synthesis of cytosolic sterols and mitochondrial ubiquinone [118]. This latter pathway uses G3P and pyruvate to synthesize IPP.

Additionally, phosphoenolpyruvate carboxylase (PEPC) catalyzes the synthesis of OAA from PEP, which subsequently enters the TCA cycle, where it can be used to synthesize amino acids. In *Chlamydomonas*, PEPC complexes are represented by two subtypes, PEPC1 and PEPC2 [119]. Multiple studies have reported PEPCs as cytoplasmic enzyme complexes [120,121].

PEP production by glycolysis occurs in both the cytosol and chloroplasts of *Arabidopsis* [122], whereas the glycolytic pathway in green microalgae such as *Chlamydomonas reinhardtii* is not duplicated in the two compartments, but different parts of the pathway are localized to different subcellular compartments. The upper half of glycolysis (glucose to 3-PGA) in *Chlamydomonas* takes place in the chloroplast, whereas the lower half (3-PGA to pyruvate) is confined to the cytosol [123,124]. Enolase, or phosphopyruvate hydratase, is the glycolytic enzyme that catalyzes the conversion of 2-phosphoglycerate (2-PG) to PEP and water [125]. Enolase, phosphoglycerate mutase [124,126], and pyruvate kinase are all absent from algal chloroplasts [126]. The PEP generated in glycolysis can play a crucial role in gluconeogenesis. Gluconeogenesis serves as a key pathway for integrating sugar, organic acid, amino acid and nitrogen metabolism, and lipid production and impacts developmental processes and the regulation of metabolite concentrations in different subcellular compartments.

*Arabidopsis* harbors three enolase variants, whereas the genome of *Chlamydomonas* harbors a single isoform. Enolase appears to be part of a large complex that represents a phosphoglycerate mutase–enolase metabolon that is critical for the colocalization of mitochondria and chloroplasts in plants [127]. The PEP carboxykinase (PEPCK) reaction in chloroplasts catalyzes the reversible decarboxylation and phosphorylation of OAA to generate PEP and CO_2_. *Chlamydomonas* has two PEPCK isoforms, with PEPCK1 predicted to be in the chloroplast and PEPCK2 in the cytoplasm [128]. These enzymes also contribute to the maintenance of PEP and OAA levels in response to the cell’s demands for these and downstream metabolites [129].

As indicated above, the occurrence of PEP in the chloroplast is essential to support vital biosynthetic processes. *Chlamydomonas* synthesizes PEP through the enzymatic activity of chloroplast PEPCK, with additional PEP synthesized by the enolase reaction of glycolysis in the cytosol. The activity of PPT would be required to facilitate the transport of PEP across the chloroplast envelope to help control its cellular distribution. *Arabidopsis* has two *PPT* genes encoding PPT1 and PPT2; both are chloroplast localized. These genes belong to distinct subgroups and have deduced protein sequences that are 52% identical at the amino acid level. Despite the divergence of these isoforms, both transporters exhibited similar substrate specificities, allowing them to move PEP across the chloroplast envelope. However, these *PPT* genes display different tissue-specific expression patterns. At*PPT1* is predominantly expressed in leaves and root vasculature and especially in xylem parenchyma cells but not in leaf mesophyll cells. In contrast, At*PPT2* is ubiquitously expressed in leaf tissue but not in roots [94]. Given the absence of identified PPTs in *Chlamydomonas* and the requirement for this activity to sustain algal metabolism, it seems likely that PEP is the preferred substrate of at least one of the uncharacterized *Chlamydomonas* sugar-P transporters, which emphasizes the need for experimentally determining transporter substrate specificities.

#### 4.2.5. GP Transporters

During periods of increased sink demand or at night when photosynthesis does not directly provide energy for metabolism, the turnover of the cell’s starch reserve appears to serve as a major source of carbon backbones and energy. Starch breakdown in the chloroplast results in the release of maltose and glucose, which can be exported from chloroplasts [130] when required in other compartments of the cell. The transport of both maltose and glucose across the plant chloroplast envelope is facilitated by the maltose transporter MEX1 and the putative glucose transporter pGlcT, respectively [131,132].

In non-green plant tissues, non-pigmented plastids depend on the import of organic carbon in the form of Glc6P by the Glc6P/Pi transporter (GPT) [81] to sustain plastid metabolism. The GPT accepts Glc6P, triose-P, 3-PGA, and xylulose 5-phosphate (Xul5P) as counter-exchange substrates for Pi [81]. *Arabidopsis* has two GPT isoforms (GPT1 and GPT2) with similar transport properties. Baune et al. (2020) suggested that GPT1 is essential in both plastids and peroxisomes, while GPT2 is specifically targeted to plastids [133]. GPT1 is crucial to sustain the oxidative pentose phosphate pathway and fatty acid biosynthesis, and *Arabidopsis* mutants lacking GPT1 exhibit defects in fertilization and seed set. However, the absence of GPT2 did not have an apparent impact on the growth and development of *Arabidopsis* plants under greenhouse conditions. Double mutants defective for both *GPT1* and *GPT2* were lethal [133,134].

Interestingly, *Chlamydomonas* expressing the gene encoding the plasma membrane hexose uptake protein 1 (HUP1) from *Chlorella kessleri* can grow on a medium containing hexoses, although slowly [19]. This result suggests that glucose (and/or metabolites generated from glucose) can be imported from the cytosol into chloroplasts, where it is metabolized. Chloroplasts can import/export glucose through either a glucose (hexose) transporter (HXT) or by first phosphorylating glucose via hexokinase activity followed by the trafficking of the glucose-P by GPT. Although Terashima et al. (2011) identified hexokinase in the chloroplast and not in the cytosol, there is speculation about dual targeting of this protein [120]. Significantly, the inability of chloroplast proteomic studies to unequivocally identify HXT or GPT probably indicates that they are present at low abundance on the chloroplast envelope [126]. This is congruent with the sluggish metabolism of *Chlamydomonas* maintained on glucose. Findinier et al. [135] suggested that the *MEX* gene in *Chlamydomonas* and its *Arabidopsis* ortholog, the maltose exporter (*MEX1*), might also be able to transport glucose across the chloroplast envelope. Additionally, the *Chlamydomonas* genome harbors a gene (Cre03.g206800) with similarities to the glucose transporter of vascular plants. The specific function of this protein has not yet been determined [135].

### 4.3. Non-PT Transporters

#### 4.3.1. Introduction

Various metabolites in addition to sugar-P must be transported across the chloroplast envelope; these include malate, OAA, succinate, fumarate, amino acids, etc. Physical highways for metabolite movement play vital roles in maintaining the integration of metabolic pathways among the various subcellular compartments. The inner chloroplast and mitochondrial membranes in plants and algae harbor various di- and tricarboxylate transporters, facilitating malate transport through counter-exchange mechanisms with other organic acids [73].

#### 4.3.2. Dicarboxylate Transporters

Dicarboxylic acid transporters are present in various subcellular compartments and can have either a narrow or broad capacity for translocating substrates, although with different kinetic features [136]. These transporters promote the reciprocal exchange of different dicarboxylates, including malate, OAA, 2-oxoglutarate, aspartate, glutamate, succinate, and fumarate [136]. They generally operate as bidirectional exchange carriers with the direction of metabolite movement governed by the concentration gradients of its substrates. Their function is essential to sustain carbon, nitrogen, and energy metabolism, with regulation that suggests the coordinated movement of metabolites among cellular compartments.

The *Arabidopsis* genome contains three dicarboxylate transporters situated on the chloroplast envelope, namely DiT1, DiT2.1, and DiT2.2, as identified by Taniguchi et al. [137] and Renne et al. [136]. Additionally, three *Arabidopsis* mitochondrial dicarboxylate transporters have been identified [138]. Chloroplast DiT1, also named AtpOMT1, has been proposed to function as an OAA/malate transporter [137]. As determined in yeast, both DiT1 and DiT2.1 were able to efficiently transport OAA. DiT1 can also transport malate and 2-oxoglutarate but not glutamate. In contrast, substrates for DiT2.1 were demonstrated to be 2-oxoglutarate and glutamate (similar affinities) in exchange for malate. Renne et al. [136] proposed that DiT2.1 functions as a glutamate/malate transporter and that it may be essential for exporting glutamate from the plastid stroma to the cytoplasm of the cell. Among the *dit* mutants, *dit2.1* is the only mutant that exhibited a photorespiratory phenotype with the growth of *Arabidopsis* seedlings defective at ambient CO_2_ when grown in a 12 h light/dark cycle [136].

Over 35 years ago, a two-translocator model linked to malate transport was proposed [139], suggesting the trafficking of 2-oxoglutarate and glutamate to assist in the assimilation of ammonium in plant plastids. In this model, the spinach dicarboxylate transporter, DiT1, would facilitate the import of 2-oxoglutarate into chloroplasts in counter-exchange with malate. The GS (Glutamate Synthase)/GOGAT (Glutamate Oxoglutarate Aminotransferase) enzymatic coupling would then convert the 2-oxoglutarate to glutamate, with the DiT2 translocator facilitating the export of the glutamate to the cytosol, again in counter-exchange with malate. The coordinated interplay of DiT1 and DiT2 ensures that the import of 2-oxoglutarate and the export of glutamate are maintained with net-zero malate trafficking.

*Arabidopsis* mitochondrial dicarboxylate transporters, DIC1, DIC2, and DIC3, are members of the mitochondrial carrier protein family. These translocators facilitate the transport of a range of dicarboxylic acids, including malate, OAA, succinate, maleate, and malonate, in exchange for phosphate, sulfate, and thiosulfate. However, these transporters were not able to efficiently use 2-oxoglutarate [138]. Given the wide-ranging ability of DICs to transport dicarboxylates, they could potentially contribute to several crucial metabolic functions that involve the flux of organic acids and reducing equivalents to or from mitochondria. These processes encompass primary amino acid synthesis (ammonium assimilation), fatty acid metabolism (mobilization during seed germination), gluconeogenesis, isoprenoid biosynthesis, nitrate assimilation, and the export of mitochondrial redox equivalents to the cytosol [140]. Recently, it was demonstrated that DIC2 serves as a mitochondrial malate/citrate counter exchanger in *Arabidopsis* leaves [141]. The translocation of these metabolites plays a role in regulating the import of malate into mitochondria, which can then serve as a substrate for respiratory metabolism and oxidative phosphorylation. Within the mitochondria, the malate is converted to citrate, which can either be used to sustain the TCA cycle, be exported to fuel cytosolic biosynthetic pathways, or be stored in vacuoles [141].

#### 4.3.3. Oxaloacetate/Malate Transporters

Malate trafficking among compartments of photosynthetic eukaryotes indicates a critical role of this metabolite for sustaining TCA cycle activity, the C4-dicarboxylic acid, and the glyoxylate cycle. Malate, synthesized through the reduction of OAA by MDH, is a dual provider of carbon backbones and reducing equivalents to various cellular/subcellular compartments. Generally, most reducing equivalents in photosynthetic cells are trafficked through the movement of reduced metabolites, with malate and triose-P being the dominant mobile carriers [57,58,127]. The malate shuttle requires the coordinated movement of malate across organelle membranes by specialized dicarboxylate transporters and the activity of MDHs; the latter functions in multimeric complexes [142,143]. Malate/OAA antiporters (OMTs) are among the most well-studied dicarboxylate transporters, exchanging OAA and malate across the chloroplast and mitochondrial envelopes.

The *Arabidopsis* genome contains nine genes encoding MDHs. Two of the MDHs are located in the chloroplast, two in the mitochondria, two in the peroxisome, and three in the cytosol [143]. The *Chlamydomonas* genome encodes five MDH isoforms: two in the chloroplast, MDH1 [144] and MDH5 (unpublished); two in the mitochondria, MDH2 and MDH3 [144]; and MDH2 in the peroxisome [145]. Depending on the nature of the MDH isozyme, it can use various forms of reductant (e.g., NADH, NADPH or reduced FDX); the specificities for the substrates are determined by the properties of the specific MDH isozymes. Malate also serves as a substrate for the malic enzyme (MME), which catalyzes malate decarboxylation, generating pyruvate, CO_2_, and NAD(P)H + H^+^. The generation of NAD(P)H by MME has been shown to be a primary source of reducing equivalents for fatty acid synthesis in *Arabidopsis* [146] and *Phaeodactylum tricornutum* [147].

#### 4.3.4. Tricarboxylate Transporters

The tricarboxylate transporters, part of the mitochondrial carrier protein family (not identified in chloroplasts so far), have been recognized in various organisms, including plants. These transporters can facilitate the exchange of both dicarboxylates and tricarboxylates across the mitochondrial membrane. In *Arabidopsis*, one gene encoding a Dicarboxylate-Tricarboxylate Carrier (DTC) has been identified. This carrier is proficient at translocating dicarboxylates (e.g., 2-oxoglutarate, OAA, malate, and succinate) and tricarboxylates (e.g., citrate, isocitrate, cis-aconitate, and trans-aconitate) using a counter-exchange mechanism and, like dicarboxylate transporters, may play important roles in supporting various metabolic processes: primary amino acid synthesis, nitrate/ammonium assimilation, export of reducing equivalents (e.g., for photorespiration), fatty acid metabolism (e.g., lipid mobilization and fatty acid elongation), gluconeogenesis, and isoprenoid biosynthesis [148].

#### 4.3.5. *Chlamydomonas* Putative Dicarboxylate and Tricarboxylate Transporters

In a review of the literature, Dao et al. [72] indicate that the *Chlamydomonas* genome encodes three potential plastid 2-oxoglutarate (or OAA)/malate transporters identified as OMT1, OMT2, and low-carbon-inducible20 (LCI20), along with one mitochondrial transporter designated MiTC14. BlastP searches for *Arabidopsis* and *Chlamydomonas* di- and tricarboxylate transporter proteins identified *Chlamydomonas* plastid OMT1 and OMT2 as homologues of *Arabidopsis* DiT1. Additionally, they suggest that LCI20 in Chlamydomonas could be the homologue of DiT2.1 and DiT2.2. Finally, *Chlamydomonas* MiTC14 was suggested to be homologous to either DIC1, DIC2, DIC3, or DTC in *Arabidopsis* [72]. Mitochondrial translocators that use pyruvate, fumarate, succinate, and amino acids as their substrates have been identified and studied in various eukaryotic systems [reviewed by [74,149]]. Each of these transporters plays significant roles in maintaining/regulating cellular metabolism in response to dynamic biotic and abiotic conditions. However, due to a lack of comprehensive information regarding the identification and characterization of these transporters in *Chlamydomonas*, this sparsely explored realm warrants consideration for future investigations.

## 5. Biological Processes and Metabolite Transport

### 5.1. Overview

Various processes in photosynthetic organisms require extensive movement of metabolites among subcellular compartments and the integration of spatially separated components of a pathway. Sustaining this network of cellular metabolisms means that specific compounds can be partially metabolized or transformed in one compartment of the cell and then trafficked to another for additional processing. The constant production and the dynamic partitioning of cellular metabolites, with coordination between the use and production of these metabolites, and their connectivity with various cellular processes require regulatory interactions that can reflect the accumulation of specific metabolites, the generation of ROS, and changes in the intracellular/intraorganellar redox status. Examples of critical physiological processes that require multiple reactions in various subcellular compartments and the movement of metabolites among compartments include the CO_2_-concentrating mechanism (CCM), photorespiration, fermentation metabolism, replenishment of biosynthetic pathways with depleted metabolites, and the biosynthesis of lipids; we highlight some of these processes below but, first, discuss Rubisco and its evolutionary features that are relevant to the processes described.

### 5.2. Early Evolution of Rubisco

The most abundant protein on the planet, Rubisco, occurs in plants, algae, archaea, and various bacteria (e.g., cyanobacteria) and serves a critical role in fixing CO_2_. Plants and algae have a Form 1 Rubisco that is assembled from eight small and eight large subunits to form a hexadecamer [150]. Other forms of Rubisco such as Form II are present in proteobacteria, archaea, and dinoflagellates, whereas Form III is present in archaea. Forms II and III Rubisco are more closely related to each other than to Form I.

During the early history of life on Earth (>2.5 Bya), Rubisco experienced very high levels of atmospheric CO_2_ and essentially no O_2_, resulting in the evolution of Rubisco with a low affinity for CO_2_ and the ability to catalyze an oxygenation reaction, which is energetically wasteful [151,152,153]. The early Earth environment favored Rubisco carboxylation because of high atmospheric CO_2_ and near-zero O_2_ levels; however, as photosynthesis spread across the planet, atmospheric CO_2_ declined as this molecule was transformed into organic compounds, and atmospheric O_2_ levels rose as the result of photosynthetic ‘water splitting’; these elevated O_2_ levels competitively inhibit CO_2_ fixation by Rubisco.

Rubisco oxygenation is the initial step in the process of photorespiration, which not only depresses CO_2_ fixation but also results in the synthesis of 2-phosphoglycolate, a potentially toxic metabolite. Photorespiration has become prominent in the Earth’s low-CO_2_ atmosphere, especially at elevated temperatures [154]. Additionally, the K_1/2_ of Rubisco for CO_2_ is high (low substrate affinity), and diffusion from the atmosphere into an aquatic environment and across the membranes of living cells is slow. As a means of combatting the highly suboptimal kinetic characteristics of Rubisco under our current atmospheric conditions, a variety of strategies evolved to concentrate Ci within cells and in the immediate neighborhood of Rubisco.

### 5.3. The CCM

The CCM comes in various ‘forms’, including both a biochemical and biophysical CCM. Both cyanobacteria and many algae, including green algae and diatoms, have a biophysical CCM in which Ci is routed to the site of CO_2_ fixation. C4-type plants [155] use a biochemical CCM that relies on the movement of CO_2_ and reductant via organic acids. In contrast, C3 plants, which include many crop plants, are unable to concentrate Ci.

#### 5.3.1. C4 Metabolism and Biochemical CCM

C4-type plants have both mesophyll and bundle sheath cells. Generally, the CCM associated with these plants involves the carboxylation of PEP in the cytoplasm of mesophyll cells by PEP carboxylase to generate OAA, which moves into mesophyll cell chloroplasts, where it is reduced to malate. The malate moves out of mesophyll chloroplasts and into chloroplasts of bundle sheath cells where it is decarboxylated and converted to pyruvate, CO_2_, and NADPH by the MME, and both the CO_2_ and reducing equivalents generated are concentrated around Rubisco, leading to efficient carboxylation and the generation of two molecules of 3-PGA, which are reduced to G3P. The pyruvate generated by the MME reaction can be transported back to mesophyll cells, where it can move into chloroplasts and be used to regenerate the PEP that sustains the C4 metabolism. This process requires trafficking of several metabolites between cells and among cellular compartments, which involves various transporters of organic acids on the chloroplast envelope of mesophyll and bundle sheath cells; a simplified version of the trafficking of metabolites in C4 photosynthesis is depicted in Figure 3A.

#### 5.3.2. Biophysical CCM

Cyanobacteria and algae can have a biophysical CCM, which relies on the trafficking of Ci among different subcellular compartments. Proteins that comprise core components of algal and cyanobacterial CCMs include bicarbonate and CO_2_ transporters and a range of carbonic anhydrases (CAHs) that are specifically positioned in the cell, where they catalyze rapid HCO_3_^−^-CO_2_ interconversion. The cyanobacterial compartment that houses Rubisco is designated the carboxysome; a functionally, but not physically, analogous compartment in many (but not all) algae is called the pyrenoid. The carboxysome is delimited by a proteinaceous shell and, in addition to Rubisco, houses specific linker proteins associated with Rubisco and CAH activities. Under low-Ci conditions, most CO_2_ fixation in cyanobacteria occurs in carboxysomes. Its proteinaceous shell likely limits CO_2_ leakage [reviewed in [159]]. Plasma membrane Ci transporters (both CO_2_ and HCO_3_^−^ transporters) deliver Ci to the cytoplasm in the form of HCO_3_^−^. The HCO_3_^−^ diffuses into the carboxysome where it is rapidly converted to CO_2_ by a carboxysome-associated CAH. The released CO_2_ is then fixed by Rubisco and Calvin-Benson cycle enzymes. Some of the components involved in carboxysome biogenesis appear to be induced under low-CO_2_ conditions. The NDH-1_3_ and NDH-1_4_ complexes are believed to function in capturing CO_2_ that diffuses across the plasma membrane or leaks out of carboxysomes into the cytosol by converting it to HCO_3_^−^ via a unidirectional hydration of CO_2_ to HCO_3_^−^ that is coupled to PET [160]. In β-cyanobacteria, NDH-1_3_, which has a high affinity for its substrate, is induced under limiting Ci conditions, whereas the NDH-1_4_ lower-affinity transporter is constitutively expressed [161,162].

In green algae like *Chlamydomonas*, the CCM is accompanied by the development of a ‘mature’ pyrenoid in the chloroplast stroma [reviewed in [163]]. This intrachloroplast liquid–liquid structure is packed with Rubisco, surrounded by a starch sheath, and traversed by thylakoid membranes. Under low-CO_2_ conditions, the lumenal α type CAH, CAH3 is concentrated in the thylakoid membranes that penetrate the pyrenoid matrix, where it helps supply Rubisco with CO_2_. The starch sheath that surrounds the pyrenoid may be comparable to the carboxysome shell, limiting the leakage of CAH3-generated CO_2_. Overall, pyrenoid development and other low-CO_2_-associated processes, such as mitochondria repositioning to the cell periphery [164], the induction of genes encoding putative HCO_3_^−^ transporters that are targeted to the plasma membrane [165], the inner envelope membrane of the chloroplast [165], and the thylakoid membranes [166], and the routing of HCO_3_^−^ to pyrenoid-localized thylakoids where it can be converted to CO_2_ by CAH3 (Figure 3B), provide Rubisco with high levels of its substrate [165], which increases the cell’s photosynthetic efficiency.

Several CCM components have been studied in *Chlamydomonas*; the positions of these proteins in the cell and movement of Ci to its site of fixation are depicted in Figure 3B. The *Chlamydomonas* chloroplast envelope transporter, LCIA, examined by Förster [167], is part of the NAR1 family of nitrite/formate transporters. Both LCIA expression and the phenotype of a *lciA* mutant strongly indicate a role for this transporter under low/very low CO_2_ and when HCO_3_^−^ is the main Ci form available for uptake (e.g., at elevated external pH). The expression of *LCIA* in *Xenopus* oocytes [168] demonstrated the ability of this transporter to use HCO_3_^−^ as a substrate. Additionally, both the *E. coli* and Arabidopsis *cah* mutants (unable to grow at ambient CO_2_) were exploited to measure the capacity of LCIA to rescue the growth phenotypes of these strains [167]. LCIA, targeted to the plasma membrane of the *E. coli* mutant and the chloroplast envelope of the *Arabidopsis* mutant, enabled substantial growth of these strains under ambient CO_2_ conditions. Based on the silicone oil centrifugation assay [169], LCIA was also shown to directly improve bicarbonate uptake in *E. coli* [167]. Additionally, the CAH enzymes βCA1 and βCA5 of *Arabidopsis* participate in chloroplast carbon metabolism by supplying HCO_3_^−^ to the enzyme acetyl-CoA carboxylase, the first catalytic step in the synthesis of fatty acids. A mutant of *Arabidopsis* in βCA5, which is mostly expressed in roots, exhibited severely impaired growth at ambient CO_2_ levels [170]. This mutant could be rescued by the *Chlamydomonas* LCIB protein, suggesting that LCIB has CAH activity [171].

The impact of LCIA expression on Ci utilization has been examined in various plants. Nolke et al. (2019) reported that the expression of LCIA or the *Chlamydomonas* CAH3 in tobacco improved plant growth [163,172], whereas Atkinson et al. [168] did not observe an effect of the expression of LCIA-GFP when localized to the chloroplast envelope of wild-type *Arabidopsis*. Förster et al. [167] evaluated several photosynthetic parameters in tobacco plants expressing LCIA-GFP but also did not observe enhanced fitness. A general conclusion based on these experiments is still ambiguous, as the results not only reflect expression levels of the LCIA-GFP constructs but also the ability of the introduced protein to find its proper subcellular location and disposition in the membranes. Additionally, various proteins may require specific post-translational modifications to retain their activity and integrate with activities of other plasma membrane and thylakoid transporters; such modifications and interactions might not readily occur when a protein is expressed in a heterologous system.

The studies discussed above suggest that in certain instances, bacteria, yeast, and plant knockout strains can be used to assay the functionality of potential HCO_3_^−^ transporters and CAHs (as well as other transport proteins), although the results may require cautious interpretation because various transporters can use more than one substrate, may not be expressed at ‘physiological’ levels, and may not experience efficient, optimal, intracellular targeting. Ten years ago, it was proposed that increasing the stromal Ci concentration could improve the biomass yield in crop plants by as much as 9% [173], potentially by inserting an active HCO_3_^−^ transporter into the chloroplast envelope and/or introducing a CAH to the stroma, which would trap CO_2_ as HCO_3_^−^. A recent model of the *Chlamydomonas* CCM suggests that the introduction of stromal components of the CCM (pyrenoid components + stromal CAH) into plants could potentially facilitate more efficient Ci utilization [174]. The introduction of a highly active stromal CAH that couples with the generation of a Ci gradient and the fixation of CO_2_ might promote HCO_3_^−^ diffusion across the envelope-localized LCIA channel. Initial improvements in the yield of biomass production in plants could then be tuned for optimal performance.

### 5.4. Photorespiration

Photorespiratory metabolism, depicted in Figure 3C, is associated with the catalytic properties of Rubisco and manifest under high-light and low-CO_2_ conditions. It is considered energetically wasteful because it produces one molecule of 3-PGA at a lower rate than occurs for Rubisco carboxylation, and one molecule of 2-phosphoglycolate while releasing a molecule of CO_2_. The reductive carboxylation pathway expends three molecules of ATP and two of NADPH per RuBP regenerated, incorporating a single carbon into hexose [175]. In contrast, the oxygenation reaction expends 3.5 ATP and 2 NADPH per RuBP regenerated but does not result in the fixation of CO_2_ [176,177]. The photorespiratory loss of fixed carbon as CO_2_ has a significant energetic cost to the cells and favors nitrogen release in the form of ammonia, which could potentially attain concentrations that are detrimental to cell growth and metabolism. Additionally, the 2-phosphoglycolate produced is potentially toxic.

The initial step of photorespiration involves the Rubisco-catalyzed oxygenation of RuBP and the formation of 3-PGA and phosphoglycolate. Phosphoglycolate phosphatase (PGP) converts 2-phosphoglycolate to glycolate and Pi in the chloroplast [178]. Glycolate can be used as a carbon source for the photosynthetic carbon oxidation cycle, which, together with N and S assimilation and C1 metabolism, generates essential amino acids and intermediate metabolites. Sustaining cell viability under conditions that favor photorespiration requires extensive trafficking of metabolites among cell compartments (Figure 3C).

Acclimation to limiting CO_2_ coincides with dramatic changes in photorespiratory metabolism; as the CO_2_/O_2_ ratio decreases, the oxygenation reaction of Rubisco becomes more prevalent, producing more phosphoglycolate, which inhibits triose-phosphate isomerase and interferes with the regeneration of RuBP. Physiological, biochemical, transcriptomic, and metabolomic studies all indicate a rapid and transient increase in photorespiratory metabolism shortly after shifting *Chlamydomonas* from high to low CO_2_, but prior to CCM induction [179,180,181,182,183,184,185,186]; CCM activity would diminish the level of photorespiratory metabolism by concentrating CO_2_ in close proximity to Rubisco, as discussed. Additionally, the PGP activity increases to a maximum at 3–5 h after transferring *Chlamydmonas* cells to low-CO_2_ conditions, although expression of the three *PGP* genes (*PGP1*, *PGP2* and *PGP3*) on the *Chlamydomonas* genome remained unchanged [185,187]. However, other reports indicate that *PGP1* expression/enzyme activity is similar to that of other low-CO_2_-regulated genes [180,187]. Presumably, the PGP activity present even when CO_2_ levels are not limiting allows for rapid dephosphorylation of the toxic phosphoglycolate molecule in response to a sudden change in the environmental CO_2_/O_2_ ratio (when the ratio decreases), with the potential to export accumulated glycolate. However, there are transient increases in transcripts encoding several *Chlamydomonas* photorespiratory proteins over the time required for CCM induction [180,187]. Genes encoding alanine amino transferase (AAT1) [188], glycerate kinase (GLYK), glycolate dehydrogenase (GYD1), hydroxypyruvate reductase1 (HPR1), serine glyoxylate aminotransferase1 (SGA1), serine hydroxymethyltransferase (SHMT) [189], all glycine decarboxylase subunits except the glycine cleavage system H-protein (*GCSH*), and dihydrolipoyl dehydrogenase1 (*DLDH1*) display elevated expression (accumulation) within hours of shifting cells from high to low CO_2_ [185]. These genes appear to be regulated by CO_2_ levels and the CIA5 regulatory factor, which is considered the CCM master switch [180,187]. Metabolomic studies confirmed an increase in pool sizes of photorespiratory pathway intermediates, including glycine, glycerate, glyoxylate, serine, and 3-PGA [184], following the transition of *Chlamydomonas* cells to low-CO_2_ conditions. Metabolite levels increased as early as 30 min after transferring cells to low CO_2_, but they returned to their initial levels within 24 h of the transfer, after CCM activity developed, which exposed Rubisco to elevated CO_2_ levels [at least 20X ambient CO_2_ [169]], thereby suppressing oxygenase activity. Interestingly, the *pgp1* and *gdh1* mutants require high CO_2_ for normal growth, suggesting that the CCM does not completely suppress Rubisco oxygenase activity and photorespiratory metabolism [190,191].

Land plants and *Chlamydomonas* differ in the compartments used to further process glycolate. In plants, the glycolate generated in the chloroplast is oxidized to glyoxylate in the peroxisome via glycolate oxidase, which uses O_2_ as an electron acceptor, forming H_2_O_2_. The H_2_O_2_ is converted to H_2_O and O_2_ by catalase, and the glyoxylate is further metabolized in the peroxisome and the mitochondria to glycine and serine [175,192,193]. *Chlamydomonas* oxidizes glycolate to glyoxylate, utilizing a GYD located in mitochondria, donating the reductant generated to the ubiquinone pool of the respiratory electron transport chain, which avoids the formation of H_2_O_2_ [190] (Figure 3C). Glyoxylate metabolism in the mitochondria proceeds along the same fundamental pathway as that determined for plants.

Much of the past work on Rubisco and photorespiration has focused on improving the specificity of Rubisco for CO_2_ relative to O_2_, although the approaches that have been attempted generally result in a slower rate of substrate turnover [194,195]. Recently, potential strategies for increasing crop yields using bioengineering strategies have focused on bypassing the photorespiratory metabolism, which could potentially increase both photosynthetic efficiency and the production of biomass [196,197,198].

### 5.5. Fermentation Metabolism (Hypoxia, Anoxia)

In the dark, under oxic conditions, *Chlamydomonas* can take up and use acetate as an exogenous source of fixed carbon. The acetate can be ligated to -CoA by acetyl-CoA synthetase and enter metabolism via the glyoxylate cycle/shunt [199,200]. This shunt is a variation of the TCA cycle and occurs in peroxisome microbodies, where, in both plants and *Chlamydomonas,* β-oxidation occurs [201,202]. While many microbodies in algae and plants can look similar [203,204], the contents of the peroxisome can be both variable and dynamic [200]. The glyoxylate shunt, present in specialized peroxisomes, has five of the eight enzymes of the TCA cycle; it is missing the oxidative decarboxylating enzymes isocitrate dehydrogenase and α-ketoglutarate dehydrogenase and uses isocitrate lyase and malate synthase to bypass those reactions. This change allows the alga to metabolize fatty acids and two-carbon compounds like acetate to generate OAA without losing fixed carbon as CO_2_. The OAA can serve as a precursor for gluconeogenesis and starch production.

When *Chlamydomonas* is maintained in the dark under anoxic/hypoxic conditions, it performs fermentation metabolism [158,205]. The ability of *Chlamydomonas* to perform fermentation during acclimation to dark anoxia has been explored for over 40 years [206,207,208]. When cells experience such conditions, they cannot generate ATP by oxidative phosphorylation and, when placed in the dark, cannot perform photophosphorylation. However, anoxic/hypoxic dark-maintained *Chlamydomonas* cells can be sustained by fermentation, a metabolism in which stored carbon/starch can be degraded to Glc6P, which can subsequently be metabolized by glycolysis. Under such conditions, glycolysis metabolizes Glc6P to pyruvate, which allows for substrate-level phosphorylation. This pathway generates two molecules of NAD(P)H [synthesized by the GAPDH reaction] and two molecules of ATP (Figure 3C); the former must be reoxidized to sustain glycolysis and energy production under hypoxic/anoxic conditions. This reoxidation most commonly involves the reduction of small organic molecules, generating reduced organic molecules (see below) that may be made in both chloroplasts and mitochondria, exported from the organelles and excreted from cells.

A suite of fermentation reactions is associated with both aquatic and terrestrial photosynthetic organisms. The products synthesized are especially prominent in the dark when no O_2_ is generated by photosynthetic activity, and the exchange between terrestrial and atmospheric O_2_ can be slow; the heterotrophs consume O_2_, whereas in the dark the photosynthetic microbes are unable to help replenish the consumed O_2_, leading to rapid O_2_ drawdown. Natural ecosystems are highly dynamic, and nutrient (e.g., nitrogen, sulfur, phosphorous, iron) availability may vary and strongly impact cellular metabolism and intracellular and extracellular levels of O_2_ [209,210]. To cope with the challenges encountered in environments with highly dynamic O_2_ levels, algae have evolved a diversity of fermentation reactions [211,212,213]. Indeed, *Chlamydomonas* has been especially useful as a model eukaryotic alga to explore the metabolic, kinetic, and regulatory features of fermentation [206,207,208,211,213,214,215,216]. This alga undergoes mixed-acid fermentation, generating multiple metabolites that are synthesized in chloroplasts and mitochondria and excreted from cells; these include formate, acetate, and ethanol, generally in a ratio of ~2:1:1 [206,208,212] (Figure 3D). Fermentation in *Chlamydomonas* can also lead to CO_2_ and H_2_O production, but these components are generally synthesized at very low rates [207,208].

Two key enzymes in *Chlamydomonas* are pyruvate formate lyase (PFL1) and pyruvate ferredoxin oxidoreducatase (PFR1) (Figure 3D). PFL1 is present in both the chloroplast and mitochondria (dual targeted), where it can convert pyruvate to formate plus acetyl-CoA; its presence in both organelles may help sustain fermentation for a longer interval as the cells transition between anoxic and oxic conditions [158,217]. PFR1 is only present in chloroplasts where it generates acetyl-Co, CO_2_ and H_2_; the H_2_ is generated through the reduction of protons by hydrogenases (using electrons produced by the PFR1 reaction) and would be released from the cells as a gas. The acetyl-CoA made in both the PFL1 and PFR1 reactions can be converted to ethanol or acetate in the chloroplast. It is not clear whether ethanol can be synthesized in the mitochondrion.

Pyruvate, the substrate for both the PFL1 and PFR1 reactions, can likely be trafficked between chloroplasts and mitochondria, potentially balancing its accumulation in these organelles, although pyruvate transporters have not been identified in *Chlamydomonas*. Additionally, the induction of fermentation can be complex, with the fermentation metabolism in *Chlamydomonas* over the diel cycle occurring even under oxic conditions to meet specific energy requirements of the cells [218].

Other chlorophyte algae also use an array of different fermentative pathways, in which glycerol and lactate can be abundant products [208,212,219]. Glycerol and lactate were reported as fermentation products synthesized by some *Chlamydomonas* strains, especially under low-pH conditions (<5.5) [206,208] or by mutants in specific branches of fermentation metabolism [158,205,217]. Furthermore, the excretion of a broad range of reduced fermentative energy carriers (organic acids, alcohols and H_2_) delivers reducing equivalents and carbon substrates to the extracellular environment that can fuel the growth of coexisting heterotrophic microbes. The types and amounts of fermentation products excreted by photosynthetic microorganisms will likely impact the types and densities of organisms of the biota of various ecosystems; heterotrophic neighbors may also impact the types and levels of fermentation products synthesized by the photosynthetic microbes.

Biochemical and molecular strategies have been pursued to characterize enzymes associated with fermentation, to localize them to subcellular compartments, and to generate specific lesions in genes encoding these enzymes to help define their functions and how they are integrated among fermentation pathways. Many of these studies have been surprising, since some of the loss-of-function mutants that eliminated specific branches of fermentation metabolism showed a marked rerouting of metabolites that were not noted in the parental strains under identical conditions. Overall, these results suggest strong integration of fermentation pathways with regulatory networks that can be compensatory and redundant to accommodate the impact that environmental conditions may have on the activities of the different pathways. Dissecting fermentation metabolism, its integration across cell compartments, and the acclimation of algae to anoxia/hypoxia are critical for developing a comprehensive understanding of net carbon cycling, energy budgets, metabolic dynamics, and the potential impacts of climate change on the distribution and productivity of photosynthetic organisms in the environment.

## 6. Evolution of Transporters

Approximately 1.6 billion years ago, a cyanobacterium entered into an endosymbiotic relationship with a protist, which led to the evolution of photosynthetic organelles/plastids and the establishment of three algal lineages, glaucophytes, red algae, and green algae [220]. During plastid evolution, the integration of new transporters into the membranes of the endosymbiont played a crucial role in the export of fixed carbon from the endosymbiont (evolving organelle) to the host cell [221,222]. Studies on *Arabidopsis* and other plants have indicated that plastid sugar-P transporters evolved from a nucleotide sugar transporter (NST) of the ER and/or Golgi membranes [93] that was retargeted to the plastid inner envelope membrane [223]. This evolutionary event allowed the host to access the photosynthetic carbon pool in the form of exported nucleotide sugars [224]. Over time, the plastid NST evolved into a sugar-P transporter through changes in their substrate specificities from that of nucleotide sugars to phosphorylated sugars. These transporter specificities evolved after the divergence of Glaucophyta and other Archaeplastidae but before the split between red and green lineages [225]. Subsequent gene duplications gave rise to subfamilies of NST/plastid sugar-P transporters, such as TPTs, PPTs, and GPTs/XPTs, in the red and green lineages [80]. The Chlorophyta diverged during the early stages of Viridiplantae evolution (>1 billion years ago) to establish the Streptophyta as a separate lineage associated with streptophyte algae (Charophyta) and land plants (Embryophyta). Comparative analysis of algal plastid sugar-P mRNA sequences revealed significant differences in intron–exon organization among the different species, suggesting that they originated from gene duplications that either predated or were concurrent with early intron invasion. While the gene structure has been conserved in land plants, many algal species have additional introns that are absent in land plants. Specifically, *GPT* genes in algae contain introns acquired or lost in land plants after the Chlorophyta and Streptophyta diverged. Some introns identified in land plants are present in many, but not all, green algal species [80].

Studying the evolutionary connections among the genes encoding MDH proteins located in different cellular compartments has revealed an early divergence of two primary *MDH* genes that predates the emergence of eukaryotes. One ancestral gene led to a lineage of enzymes present in mitochondria and microbodies, whereas the second gave rise to cytosolic enzymes in plants and mammals, as well as the NADP-MDH present in chloroplasts. The plastid NADP-dependent isoform appeared early in eubacteria, before the evolution of plants. It is present in the green lineage and primitive prasinophytic algae but is absent in cyanobacteria, *Cyanophora paradoxa*, red algae, and diatoms [226,227]. Evolutionary studies of di- and tricarboxylate transporters in photosynthetic systems, however, are still scarce and represent an intriguing area for future research.

## 7. Glimpse at Regulation

### 7.1. Expression Patterns of Chlamydomonas PT and Non-PT Transporters

The various *Chlamydomomas* transporters appear to display distinct regulation at the transcript level. Here, we highlight some aspects of that regulation, based on RNA-seq analyses under a range of different environmental conditions, including over the diel cycle [104,218], nitrogen deprivation [228], and exposure to oxidative stress [229]. 

#### 7.1.1. Day/Night Cycle

As indicated in Table 1 and Supplemental Table S1, RNA-seq suggests that there are at least five different patterns of expression of genes involved in fixed carbon/reductant transport/shuttling that occur over the diel cycle. Some of these patterns show transient upregulated or downregulated transcript abundances when *Chlamydomonas* cells are shifted between the light and dark. These transient changes gradually re-establish a new level that often moves toward the pre-shift level. For other genes, the transcripts increase in abundance in either the dark or light (and may also be impacted by transient light changes). The final group of genes encodes transcripts that remain constant over the diel cycle (potentially constitutively expressed) or encodes transcripts that were not detected by the PCR-based assay. Because of their ability to transport a range of carbon compounds and redox metabolites, TPTs are believed to play a crucial role in trafficking metabolites between the chloroplast, cytosol, mitochondria, and other cellular compartments and may be responsive to the physiological conditions of the cells. Interestingly, transcripts for most TPTs either increase in the light or are regulated during transitions between light and dark. Only TPT1, TPT24, and TPT25 transcripts exhibited an increase in the dark. TPT3, TPT5, TPT6, TPT11, TPT12, TPT16, and TPT31 appear to be constitutively expressed, while the TPT23 transcript could not be detected during diel cycling. The levels of mRNAs for MDH1, MME1, MME2, MME5, and MME6 also appear to be constant over the diel cycle, whereas MME4 transcripts were not detected. Transcripts encoding mitochondrial MDHs (MDH3 and MDH4), the chloroplast MDH5, and the OMT1 and OMT2 transporters become elevated in the light, suggesting the importance of malate trafficking between chloroplasts and mitochondria during the day when photosynthesis is active. Additionally, transcripts encoding the mitochondrial CCP1 and CCP2 transporters increase in moderate-light and under very-low-CO_2_ conditions [168,230,231], although their precise physiological roles in cellular metabolism remain uncertain.

**Table 1:** Transporters and regulation of their transcript levels over the diel cycle. Raw data are taken from Strenkert et al., 2019 [218], and are presented in Supplemental Table S1. Regulation patterns are divided into five categories: transitional regulation, which is considered a short-term increase or decline in RNA levels upon transition from light to dark and dark to light phases (striped arrows); increase and decrease in mRNA levels during the dark (black arrow) or light phase (white arrow) of the cycle; and constitutive expression of a gene (black box) or no detectable expression (gray box). Upregulation and downregulation are indicated by the upward and downward directions of the arrows, respectively. The subcellular localization of the encoded proteins is noted in the last column, determined either experimentally or by in silico prediction models (Predalgo and TargetP). Abbreviations: C: Chloroplast, CE: Chloroplast envelope, Cyt: Cytoplasm, M: Mitochondria, O: Other locations (any location other than the chloroplast, mitochondrion, and secretory pathway), Pr: Peroxisome, Py: Pyrenoid, S: Secretory pathway (where proteins are directed to the translocon of the endoplasmic reticulum), G: Golgi.

		Transitional Regulation					
LocusID	Annotated Name *	Light to Dark	Dark to Light	Dark Regulated	Light Regulated	Constitutively Expressed	Subcellular Location
Cre08.g379350	TPT1		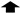				C [71]
Cre06.g263850	TPT2		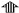				C [71]
Cre01.g045550	TPT3		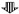				C [71]
Cre02.g106200	TPT4	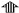			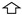		O (TargetP) S (Predalgo)
Cre02.g112900	TPT5						O (TargetP) O (Predalgo)
Cre02.g144300	TPT6		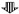				O (TargetP) O (Predalgo)
Cre03.g162000	TPT7				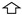		O (TargetP) S (Predalgo)
Cre03.g184850	TPT8	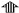					O (TargetP) O (Predalgo)
Cre04.g227450	TPT9	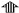					S (TargetP) O (Predalgo)
Cre07.g330850	TPT10				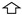		O (TargetP) O (Predalgo)
Cre08.g363600	TPT11		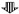				C (TargetP) O (Predalgo)
Cre09.g408400	TPT12	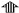	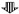				M (TargetP) C (Predalgo)
Cre09.g413700	TPT13		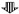		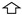		O (TargetP) O (Predalgo)
Cre08.g382350	TPT14				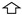		O (TargetP) O (Predalgo)
Cre09.g415900	TPT15				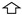		S/G [144]
Cre10.g452750	TPT16	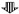	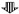				O (TargetP) S (Predalgo)
Cre11.g479950	TPT17				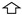		S/G [144]
Cre12.g490050	TPT18		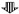		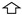		S/G [144]
Cre12.g490100	TPT19		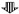		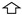		Cyt [144]
Cre12.g501000	TPT20				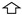		O (TargetP) O (Predalgo)
Cre15.g641266	TPT22				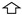		S (TargetP) S (Predalgo)
Cre15.g642950	TPT23						M (TargetP) O (Predalgo)
Cre15.g643385	TPT24			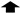			O (TargetP) S (Predalgo)
Cre16.g663800	TPT25		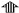	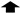			C [71] CE/Cyt [144]
Cre16.g666250	TPT26				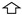		O (TargetP) M (Predalgo)
Cre17.g702700	TPT27		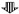		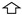		M (TargetP) C (Predalgo)
Cre17.g703250	TPT28		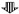		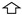		O (TargetP) O (Predalgo)
Cre17.g710850	TPT29				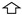		S (TargetP) O (Predalgo)
Cre11.g467754	TPT30		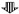		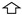		O (TargetP) O (Predalgo)
Cre14.g622700	TPT31		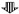				O (TargetP) S (Predalgo)
Cre18.g748947	TPT32				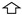		S (TargetP) S (Predalgo)
Cre09.g408428	TPT33				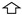		O (TargetP) M (Predalgo)
Cre03.g194850	MDH1						C/Py [144]
Cre10.g423250	MDH2			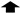			Pr [145,232]
Cre02.g145800	MDH3		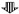		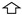		M [144]
Cre12.g483950	MDH4				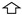		M [144]
Cre09.g410700	MDH5				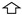		C (unpublished)
Cre06.g268750	MME1						M (TargetP) M (Predalgo)
Cre14.g629750	MME2		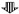				O (TargetP) O (Predalgo)
Cre14.g629700	MME3			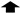			C/Cyt [144]
Cre14.g628650	MME4						M (TargetP) M (Predalgo)
Cre01.g022500	MME5						M (TargetP) M (Predalgo)
Cre06.g251400	MME6						M (TargetP) M (Predalgo)
Cre17.g713350	OMT1		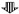		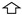		M (TargetP) C (Predalgo)
Cre17.g713200	OMT2				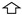		C (TargetP) O (Predalgo)
Cre06.g260450	LCI20	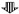	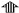				C [144]
Cre16.g672650	MiTC14	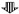	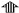				O (TargetP) O (Predalgo)
Cre04.g223300	CCP1				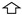		M [144]
Cre04.g222750	CCP2		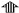		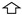		M [144]

* Sugar-P transporter family is annotated as TPT family in the v5.6 and v6.1 *Chlamydomonas* genomes.

#### 7.1.2. Nitrogen Deprivation

Transcripts for nearly two-thirds of the transporters and redox carriers presented in Supplemental Table S1 accumulated as *Chlamydomonas* cells transitioned to nitrogen deprivation conditions. This upregulation can either persist over a 48 h period of N-deprivation (length of experiment) or, after reaching a peak, decline to lower levels within that 48 h period [228]. The transcripts from the remaining genes either continuously declined or displayed an initial decline followed by a gradual increase. However, a number of TPT-type transcripts (for TPT22, TPT23, TPT24, TPT30, TPT32) were not detected following exposure of *Chlamydomonas* to nitrogen deprivation conditions. TPT13 and TPT33 transcripts exhibited little expression under nitrogen-replete conditions, with increased expression when the cells were nitrogen deprived. Among the plastid TPTs, the transcripts encoding TPT2 and TPT25 were initially diminished and then proceeded to rise. Furthermore, *Chlamydomonas* MME transcripts exhibited diminished accumulation in response to nitrogen deprivation. The MDH1 transcript, which constitutively accumulated to high levels over the diel cycle, exhibited a gradual decrease in abundance when the cells were nitrogen deprived, whereas the transcript for MDH5, the other chloroplast-localized MDH, was elevated (Supplemental Table S1).

#### 7.1.3. Oxidative Stress

As shown in Supplemental Table S1, the levels of transcripts for approximately half of the transporters and redox carriers remained unchanged when the cells were exposed to oxidative stress. For 50% of the remaining transporters/redox carriers, the transcripts became elevated, while the other 50% were depressed. Notably, all MDH transcripts, except plastid MDH5, exhibited increased abundance in response to oxidative stress.

### 7.2. Contribution of Transporters in Signaling Pathways

Photosynthesis and respiration occur in oxygen-rich environments, making both chloroplasts and mitochondria generators of ROS. O_2_ can act as an electron acceptor, and ROS can serve as regulatory molecules that elicit stress responses, ameliorating the impact of elevated electron pressure [233]. However, ROS levels must be managed by detoxification activities to maintain physiological fitness. Interestingly, ROS, redox equivalents, and sugars synthesized under high-light conditions can serve as signals that appear to coordinate nuclear and chloroplast gene expression [234,235]. Hydrogen peroxide (H_2_O_2_) is the most prevalent ROS species and can function as a redox messenger [236], participating in retrograde and anterograde signaling from various cell compartments to control nuclear gene expression [237,238,239].

As suggested from the above discussion, while sugar-P/Pi and malate/OAA shuttles participate in the transport of fixed carbon and redox equivalents, their activities also impact the regulation of signaling pathways. Various studies have demonstrated that in plants, the export of triose-P is critical for regulating nuclear gene expression when cells are exposed to high light [240,241]. Recently, Zirngibl et al. [235] reported that sugar-P export from chloroplasts by TPTs leads to a rapid increase in the cellular sugar content, which ultimately triggers transcriptional and metabolic activation of anthocyanin biosynthesis during high-light acclimation. In *Arabidopsis*, studies have shown that the reductant is trafficked from chloroplasts to mitochondria through the plastid MDH-DiT1 and the mitochondrial MDH1 pathway, which must also involve a mitochondrial dicarboxylate transporter. This process provides respiratory complex I with NADH, eventually producing ROS as a side product. This ROS is proposed to act as a signaling molecule that activates Programmed Cell Death (PCD) in the mosaic death 1 (mod1) mutant [242].

## 8. Moving Forward

Almost all cellular processes involve metabolic pathways that occur in specific subcellular compartments. In several cases, pathways in one compartment may require metabolites generated in a second compartment, which would necessitate the activities of metabolite transporters associated with the various cellular compartments. For algae and plants, there are ambiguities concerning the range of substrates used by the various transporters. In other cases, while we may know that an organelle must be able to traffic a particular metabolite, the transporters involved remain unidentified. Additionally, overlapping substrate specificities among transporters may allow for compensation among the transporters when one of the activities is lost or inhibited. Furthermore, we know little about how the levels and activities of these transporters are controlled. For example, transcripts encoding various transporters may increase when their transport activity is required [243,244,245], or the regulation of transport and metabolic activities may be at the translational or post-translational levels [246,247,248,249,250]. Various genes associated with the CCM (e.g., for Ci transport) may be activated through transcriptional control when the CO_2_ levels become depressed [183,185,251], whereas some of the fermentation enzymes may not be impacted at the transcriptional level but may be controlled at the level of translation or post-translational modifications [158].

There are aspects of metabolite trafficking and organelle interactions for which there is an extreme dearth of information. Under different environmental conditions, organelles may assume distinct positions in cells that better exploit their capabilities. For example, under very-low-CO_2_ conditions, mitochondria move from internal positions in *Chlamydomonas* cells to a cortical position [164,252], which may allow them to better deliver energy for concentrating Ci (more efficient operation of the CCM), although this still requires experimental verification. The mechanisms by which specific organelles move and rearrange their morphologies are just being explored, although both microtubules and actin are likely to play a role in the process. Additionally, metabolite transport across the chloroplast and mitochondrial envelopes may be linked to metabolic reactions that generate substrates for transport; this link between the synthesis of metabolites and their transport may also necessitate physical interactions between mitochondria and chloroplasts [253,254,255] that make the transport process more ‘directed’ and efficient. It was found that some glycolytic enzymes have moonlighting functions; phosphoglycerate mutase, enolase, and pyruvate kinase form a large complex that creates a metabolite channel and mediates the colocalization of chloroplasts and mitochondria [127]. There is still much to be learned about direct and indirect interactions among organelles and how that might facilitate the movement of metabolites between compartments and create more efficient biological processes.

## Supplementary Materials

The following supporting information can be downloaded at: https://www.mdpi.com/article/10.3390/plants13213015/s1.

## Nomenclature

AAT1	Alanine amino transferase
Acetyl-CoA	Acetyl coenzyme A
ADP	Adenosine diphosphate
AEF	Alternative electron flow
AOX	Alternative oxidase
ATP	Adenosine triphosphate
CAH	Carbonic anhydrase
CBC	Calvin–Benson Cycle
CCM	CO_2_ concentrating mechanism
CEF	Cyclic electron flow
CGL51	CONSERVED GREEN LINEAGE 51
Ci	Inorganic carbon
CMEF	Chloroplast to mitochondria electron flow
CO_2_	Carbon dioxide
CytOx	Cytochrome oxidase
DAHP	3-deoxy-D-arabino-heptulosonate 7-phosphate
DAHPS	3-deoxy-D-arabino-heptulosonate 7-phosphate synthase
DHAP	Dihydroxyacetone phosphate
DiT	Dicarboxylate translocator at chloroplast envelope
DIC	Dicarboxylate carrier at mitochondria membrane
DLDH1	dihydrolipoyl dehydrogenase1
DOXP/MEP	1-deoxyxylulose 5-phosphate/2-C-methylerythritol 4 phosphate pathway
DTC	Dicarboxylate-tricarboxylate carrier at mitochondria membrane
ER	Endoplasmic reticulum
E4P	D-erythrose 4-phosphate
FDX	Ferredoxin
FLV	Flavodiiron oxidoreductase
GAPDH	Glyceraldehyde 3-P dehydrogenase
GCSH	Glycine cleavage system H-protein
GFP	Green fluorescent protein
Glc6P	Glucose 6-P
GLYK	Glycerate kinase
G3P	Glyceraldehyde 3-phosphate
GPT	Glucose 6-P transporter
GS/GOGAT	Glutamate Synthase/Glutamate Oxoglutarate Aminotransferase
GYD1	glycolate dehydrogenase
H_2_	Hydrogen gas
HCO_3_^−^	Bicarbonate
H_2_O	Water
H_2_O_2_	Hydrogen peroxide
HPR1	hydroxypyruvate reductase1
HUP1	hexose uptake protein 1
HXT	hexose (glucose) transporter
IPP	isopentenyl diphosphate
LEF	Linear electron flow
LCIA	Low-CO_2_-inducible A
LCIB	Low-CO_2_-inducible B
LCI20	Low-CO_2_-inducible 20
MAPK	Mitogen-activated protein kinase
MDH	Malate dehydrogenase
MEX1	Maltose transporter
MFS	Major facilitator superfamily
MiTC14	Mitochondrial substrate carrier protein
MME	Malic enzyme
MVA	Acetate-mevalonate pathway
NAD(P)(H)	nicotinamide adenine dinucleotide (phosphate)(hydrogen)
NDH1	NAD(P)H-PQ reductase in plants
NDH2 (NDA2)	NAD(P)H-PQ reductase in algae
NPQ	Nonphotochemical quenching
NST	Nucleotide sugar transporter
O_2_	Oxygen gas
^1^O_2_	Singlet oxygen
O_2_^−^	superoxide
OAA	Oxaloacetate
OMT	Oxaloacetate malate shuttle
PCD	Programmed Cell Death
PCEF	Pseudocyclic electron flow
PEP	Phosphoenolpyruvate
PEPC	phosphoenolpyruvate carboxylase
PEPCK	PEP carboxykinase
PET	Photosynthetic electron transport
2-PGA	2-phosphoglycerate
3-PGA	3-phosphoglycerate
pGlcT	Putative glucose transporter
PGP	Phosphoglycolate phosphatase
Pi	Inorganic phosphate
PPT	Phosphoenolpyruvate transporter
PQ	Plastoquinone
PSI	Photosystem I
PSII	Photosystem II
PT	Phosphate transporter
PTOX	Plastoquinol terminal oxidase
PFL	Pyruvate formate lyase
ROS	Reactive oxygen species
Rubisco	Ribulose 1,5-bisphosphate carboxylase
RuBP	Ribulose-1,5-bisphosphate
SGA1	serine glyoxylate aminotransferase1
Sugar-P	Sugar phosphate
TCA	Tricarboxylic acid cycle
TPT	Triose phosphate/phosphate transporter (in *Chlamydomonas*: annotation for sugar-P/Pi transporters)
Triose-P	Triose phosphate
Xul5P	Xylulose 5-phosphate
XPT	Pentose-P or xylulose 5-phosphate transporter
